# Perceived ease of use of telehealth services and associated factors in Saudi Arabia: A cross-sectional study

**DOI:** 10.1371/journal.pone.0334943

**Published:** 2025-10-29

**Authors:** Anis Ben Ghorbal, Ibrahim Elbatal, Abdel-Rahman Aldukeel, Abdelhamid Elshabrawy, Sarah Assem Ibrahim, Niveen Ibrahim Aly El-Zayat, Samah Zakaria, Heba Ahmed Abd El-Wahab, Dina Mohsen Sabry, Thuraya Elsayed, Suzan Abdel-Rahman, Hatem Semary

**Affiliations:** 1 Department of Mathematics and Statistics, College of Science, Imam Mohammad Ibn Saud Islamic University (IMSIU), Riyadh, Saudi Arabia; 2 King Salman Center for Disability Research, Riyadh, Saudi Arabia; 3 Biostatistics and Demography Department, Faculty of Graduate Studies for Statistical Research, Cairo University, Giza, Egypt; 4 Department of Statistics, Faculty of Economics and Political Science, Cairo University, Egypt; 5 Statistics and Insurance Department, Faculty of Commerce, Zagazig University, Egypt; Shiraz University of Medical Sciences, IRAN, ISLAMIC REPUBLIC OF

## Abstract

This paper aimed to explore the impact of disability along with other factors on telehealth usage, examining the degree of ease people feel while using telehealth services in Saudi Arabia. A cross-sectional study collected data from 428 Saudi adult participants via an online survey between October and November 2024. The Extended Unified Theory of Acceptance and Use of Technology (UTAUT) model was adopted to design the questionnaire. The paper utilized the binary Logistic Regression and the random forest algorithm to predict the participants’ attitudes towards the ease of using Telehealth services. The ease of use of the telehealth system was assessed using the effort expectancy index, which measures participants’ perceptions about feasibility, clarity, simplicity, and comfort related to the usage of telehealth services. The results showed perceptions supporting the ease of using telehealth services decreased for disabled individuals by 80% (p = 0.04) compared to non-disabled individuals. In contrast, availability of facilitating conditions (OR=9.18, p < 0.001), performance expectancy (OR=4.70, p = 0.006), perceived safety (OR=3.33, p = 0.044), and social influence (OR=3.82, p = 0.016) were positively and significantly associated with perceived ease of use. The presence of perceived barriers also had a positive effect (OR=3.62, p = 0.024). The random forest algorithm outperformed logistic regression in terms of classification accuracy and AUC (0.774 versus 0.758 in the test set). Classification models indicated that factors related to telehealth technology were the most influential in perceptions of ease of use. These findings underscore the need for policymakers to develop inclusive telehealth strategies that specifically address barriers faced by disabled individuals, ensuring equitable and accessible digital health services for all.

## Introduction

According to the World Health Organization, telehealth is defined as the “delivery of health care services, where patients and providers are separated by distance. Telehealth uses information communication technology (ICT) for the exchange of information for the diagnosis and treatment of diseases and injuries, research and evaluation, and for the continuing education of health professionals”. For decades, numerous countries have been utilizing telehealth services; however, this utilization has surged during the COVID-19 pandemic. It quickly became an essential tool allowing individuals to have real time connections with healthcare providers from the safety of their homes [1]. Telehealth has proved to facilitate equitable healthcare delivery by enhancing accessibility and reducing the impediments to healthcare access [1,2]. The minimized travel requirements and faster access to medical professionals have motivated patients to have a more positive outlook on telehealth services [3]. Telehealth among adults can be considered an effective tool for accessing healthcare, particularly for routine consultations, chronic disease management, and follow-up care [4]. It has played a very successful role during the pandemic in managing patients with chronic diseases such as blood pressure. Tools such as drug interaction alerts, and blood pressure check reminders were reported to facilitate medication adherence. Patients were able to have more suitable alternatives for attending medical consultations than in-person visits which greatly reduced stress and anxiety among patients and healthcare providers [5].

Nevertheless, despite being very promising, telehealth is still facing numerous challenges. Concerns about data privacy, unreliable internet connectivity, individual and ethnic factors in addition to limited health and digital literacy were identified as barriers to broader adoption of telehealth. Furthermore, inadequate insurance coverage and reimbursement were reported to further hinder its widespread use [4,5]. The impact of these challenges is even more severe on disabled patients as they are more compromised with special social, economic and environmental limitations. These unique barriers obstruct their ability to benefit from telehealth services in the same way as patients without disability [2]. The WHO defines disability as “the result from the interaction between health conditions or impairments that a person experiences and environmental barriers that may hinder their full and effective participation in society on an equal basis with others” [1]. One of the main issues faced by disabled people in particular is the lack of proper interface design of telehealth platforms to fit their needs. For instance, many platforms are not compatible with screen readers and voice commands which makes it difficult for people with visual or motor impairment to interact with healthcare professionals virtually. Moreover, overly technical platforms can be very challenging for people with intellectual or learning disabilities. Therefore, it is essential to broaden the efforts to bridge the “digital divide” experienced by people with disability to secure inclusive access to telehealth services [1,6].

Saudi Arabia was one of the countries that had experienced substantial expansion in the usage of digital technologies to deliver healthcare services remotely [3]. This rapid development had started even before the outbreak of the COVID-19 pandemic. In fact, Saudi Arabia began exploring the possibility of telehealth usage as early as 1990. In Saudi Arabia, the Ministry of Health provides telehealth services through various platforms including virtual clinics, 937 call centers and the “Sehhaty” smartphone application [7]. There is a generally positive attitude towards telehealth, recognizing its potential to enhance healthcare access, particularly in remote and underserved regions. The importance of addressing these challenges through strategies such as improving digital infrastructure and providing training for healthcare professionals. Thus, overcoming these obstacles is critical for the effective integration of telehealth into Saudi Arabia’s healthcare system [8].

However, several obstacles have emerged in Saudi Arabia over the years. From a technological standpoint, despite the massive efforts, some isolated areas in Saudi Arabia are still facing connectivity issues. On the other hand, from a patient’s perspective, fears about the lack of personal interaction in virtual consultations and concerns related to technology usage are affecting some people’s acceptance of telehealth [3]. According to the results of the 2022 population census, 1.8% of the total population in Saudi Arabia are disabled [9]. Telehealth holds significant promise for transforming care for children with disabilities in Saudi Arabia, but its success depends on increased investment in infrastructure, training, public awareness, and supportive policy frameworks to overcome barriers and ensure effective implementation [10,11].

Several studies underscored the growing importance of telehealth in Saudi Arabia while highlighting the need for targeted strategies to overcome barriers and ensure its successful implementation [3,7,8]. While other studies focused on patients’ opinions among adults, elderly, and disabled children to address the barriers and suggest interventions [4,10–12]. Moreover, Almojaibel et al. (2025) investigated telehealth acceptance exploring the factors affecting telehealth acceptance among the Saudi population [13]. They reported that telehealth was highly accepted by the Saudi population and that the “perceived ease of use” was one of the significant factors influencing it. However, very few studies investigated the potential factors affecting the perceived ease of use (Effort expectancy) of telehealth services in Saudi Arabia.

Effort Expectancy can be analyzed through two broad approaches: theory-driven models and statistical and machine learning models. Theory-driven models are designed to explain technology acceptance, particularly intention and use behavior, like Unified Theory of Acceptance and Use of Technology (UTAUT) and Technology Acceptance Model (TAM) [14]. The statistical and machine learning models are used for predictive accuracy and variable importance ranking, like Logistic Regression (LR) and Random Forest (RF) [15,16].

Our study aims to explore the impact of disability in addition to predicting and identifying the most important determinants of perceived ease of use (Effort Expectancy) for Saudis who use telehealth. Therefore, we use statistical and machine learning models (logistic regression and Random Forest) that are better suited for achieving this aim.

## Methods

### Study design and study setting

A cross-sectional study was conducted using anonymous online surveys. Data was collected from October 1 to November 20, 2024. The target population included Saudi individuals aged 18 years or older. This study was approved by the Ethics Committee under number (00013692). All data are anonymous without any reference to the personal identity of the respondents. Participants were included if they were Saudi nationals aged 18 years or above and capable of completing the questionnaire. Individuals who were non-Saudis or younger than 18 were excluded.

### Sample size and sampling technique

A convenience sampling technique was used to recruit participants. The questionnaire was distributed via online surveys using Google Forms, which were shared on social media platforms including WhatsApp, Telegram, LinkedIn, and Facebook. The minimum required sample size was 384 Saudis, assuming 50% intention to use telehealth services, an acceptable accuracy of 5%, α 0.05 and a power of 95%. The sample size was calculated using the G-POWER program. The online survey was distributed to approximately 532 individuals, yielding 428 complete responses, corresponding to an estimated response rate of 80.5%.

### Questionnaire design

The developed questionnaire aims to investigate the use of telehealth services. The Extended Unified Theory of Acceptance and Use of Technology (UTAUT) model was adopted as the basis for designing the questionnaire [17,18]. The questionnaire covered the demographic characteristics of the individuals in terms of place of residence, age, sex, educational level, chronic comorbidities, living with a disability that prevents normal daily activity, ease of using the Internet, and previous experience with telehealth services. It also examines participants’ previous experience with these services and their views on the benefits of e-medicine, such as saving time and money (performance expectancy: PE), as well as the ease of use of the system (effort expectancy). It also examines the influence of others, such as friends and doctors, on the use of services (social influence: SI), and the user’s facilities, such as technical knowledge and resources (facilitating conditions: FC). It also discusses participants’ beliefs of their ability to complete task (Self efficacy) and participants’ feelings of safety when using the internet to transmit health information (Perceived security: PS), and potential disadvantages, such as cost and difficulty of using technology (Perceived barriers). Finally, it examines participants’ intention to use these services in the future (intention to use telehealth: IT). **The questionnaire is presented in** S1 File.

### Measures

**Fig 1:**
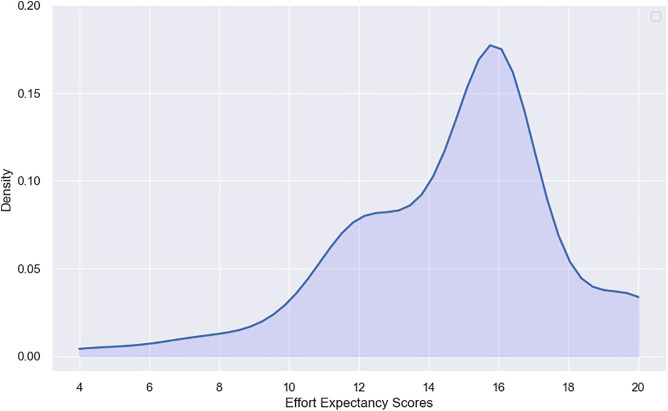
Kernel Density Plot of Effort Expectancy Scores.

The outcome variable, Effort-Expectancy (EE), is an index consisting of four components measured by 5- point Likert scale. This index consists of four components measured by 5- point Likert scale. These components reveal participants’ perceptions about feasibility, clarity, simplicity, and comfort related to the usage of telehealth services. The Effort-Expectancy Index is constructed based on their total scores. The resulting index is then discretized into a binary index, such that the cutoff point is selected based on the kernel density plot (Fig 1). Fig 1 shows a bimodal pattern, reflecting the existence of two subpopulations. The threshold 13 was chosen to vividly separate these groups, consequently, the binary index is assigned a value of 0, conforming to the label “Slightly agree”, if the total score is less than 13, otherwise, it is assigned a value of 1, corresponding to the label “highly agree”. For more information about the concept of kernel density and its associate methodology and parameters, details are provided in supplementary file 2.

Sixteen independent binary features are thought to have an influence on the target variable. They are classified as follows: (1) *Demographic factors*: sex, place of residence, age class, and educational attainment, (2) *Health-related factors*: disability, and chronic disease, (3) *Internet and eHealth usage-related factors*: Ease of net usage (easy use) and previous experience with using eHealth (Experience) and (4) *Telehealth-related indices*: Facilitating Condition (FC), Perceived Barriers (PB), Intention to Use (IU) Telehealth system, Social Influence (SI), Perceived Security, (PS), Performance Expectancy (PE), Self-Efficacy (SE), and Technology Anxiety (TA).

Each of those telehealth-related indicators is constructed from the total scores of a 5-point Likert scale of some related components. For more understanding of those indicators, it is of great importance to know that Facilitating Condition (FC) reflects the availability of technical and organizational support. Perceived Barriers (PB) represents the obstacles and difficulties in using the telehealth system. Intention to Use (IU) reflects the willingness of using telehealth services. Social Influence (SI) describes the effect of others on a person’s attitude towards using the telehealth system. Perceived Security (PS) reflects the extent of feeling secure while using telehealth services. Performance Expectancy (PE) summarizes the degree to which an individual believes that using telehealth will help them increase their health quality. Technology Anxiety (TA) refers to concerns about technology. Finally, Self-Efficacy (SE) represents a person’s belief in his ability to use the telehealth system. These factors are discretized into a binary scale using the same kernel density-based approach implemented for the “Effort Expectancy” variable.

### Statistical analysis

Since the aim of this study is to identify the most important determinants of EE, the current study uses Binary Logistic Regression (LR) and the Random Forest algorithm (RF) to predict the participants’ attitudes towards the ease of using Telehealth services. Both approaches belong to the supervised machine learning algorithms. The binary Logistic Regression, which is considered the baseline of classification tasks, assumes a linear relationship between the log of the odds of the outcomes and the covariates. However, Random Forest is a more advanced tree-based ensemble machine learning algorithm used for both regression and classification without assuming a specific model form or linear relationships. Considering both models in the current study enables us to evaluate a traditional statistical tool versus one of the ensemble machine learning approaches that gives robust results due to its ability to capture nonlinear relationships between outcome and covariates. Notably, any enhancement in predictive accuracy by RF model demonstrates the benefits of the ability of the model to capture nonlinear relationship in the data, implying more efficient and reliable decisions [15,16].

The theoretical backgrounds of both algorithms and measures of variable importance are discussed in S2 File (S.1(a) Fig, S1(b)Fig, S2 Fig). Furthermore, it demonstrates the associated accuracy measures commonly used to evaluate the models’ performance. Data is provided in S3 File, and statistical analysis codes are available in S4 File.

## Results

Data management and descriptive statistical analysis are performed using the Python statistical libraries: Pandas, Numpy, Matplotlib.pyplot, and Seaborn. The classification models are fitted and evaluated using the Python packages: Lifelines, Statmodels, Sklearn, LogisticRgression, and RandomForestClassifier. The current section describes the main characteristics of the target and features variables under study.

Table 1 represents the distribution of respondents according to their attitudes towards the degree of ease associated with using the telehealth system and their main characteristics.

**Table 1:** Characteristics of Participant by the ease of using the telehealth system “Effort Expectancy”.

Feature	Effort Expectancy Status		N (%)
	Highly agree (%)	Slightly agree (%)	
**Background Characteristics**			
**Sex**			
Female	73.1	26.9	286 (66.8)
Male	79.6	20.4	142 (33.2)
**Age class**			
< 25 (Young)	71.7	28.3	237 (55.4)
25-34 (Medium)	78.9	21.1	142 (33.2)
35+ (Old)	81.6	18.4	49 (11.4)
Place of residence*			
Rural	50	50	12 (2.8)
City	76	24	416 (97.2)
Educational level***			
High school or less	64.0	36.0	139 (32.5)
University and above	80.6	19.4	289 (67.5)
**Explanatory Variables**			
Disability status**			
Disabled	57.6	42.4	33 (7.7)
Non-Disabled	76.7	23.3	395 (92.3)
**Previous experience with** **A telehealth service**			
Experienced	77.9	22.1	136 (31.8)
Inexperienced	74.0	26.0	292 (68.2)
Easiness of using the Internet***			
Not Easy	44.1	55.9	34 (7.9)
Very Easy	77.9	22.1	394 (92.1)
**Chronic disease status**			
Have a chronic disease	69.6	30.4	46 (10.7)
Do not have a chronic disease	75.9	24.1	382 (89.3)
**N (%)**	322 (75%)	106 (25%)	428 (100%)

Chi-square test of independence significance results: ***(<0.01), **(<0.05), *(<0.1)

Table 1 shows that the majority of Saudi respondents (approximately 75%) supports the ease of using telehealth services. Further, results illustrate almost similar distribution of participants across the Effort Expectancy status for their sex, age group, previous experience with telehealth service, or chronic disease status. Besides, 44% of the respondents who considered using the Internet not easy, highly agreed that the telehealth system is easy to use. In addition, Table 1 also shows that 97% of all respondents live in the city. Moreover, 57.6% of the disabled respondents highly agreed with the ease of using the telehealth system.

Table 2 displays the rate of high agreement on the ease of using telehealth among disability subgroups across all other features. It further shows the odds ratios of supporting the ease of using telehealth, as well as its associated 95% CI, comparing disabled versus non-disabled participants stratified by multiple characteristics. In Saudi Arabia, the survey concluded that disabled are significantly 59% less likely than non-disabled to report that telehealth systems are easy to use. Results further elucidate that disabled males have significantly 83% lower odds to support easiness of using telehealth than non-disabled males. Moreover, younger disabled participants have significantly 66% lower odds of the response than non-disabled ones. However, the effect of disability is much higher for the oldest participants, since the disabled group has significantly 85% lower odds than the non-disabled group. Considering levels of education, the effect of disability on the odds of the response was only significant for participants whose education is less than secondary, where the disabled group has significantly 71% lower odds than the non-disabled group. Disabled participants who live in city have a significant decline of 61% than non-disabled. On the other hand, disabled participants with no previous experience with telehealth systems have significantly 68% lower odds than the non-disabled counterparts. Additionally, the odds ratio of disabled participants who do not find any difficulty to use the internet is significantly 55% lower than the non-disabled counterparts. Finally, the odds ratio of disabled participants having chronic disease is significantly 69% lower than non-disabled participants of the same group.

**Table 2:** Characteristics of Highly Agreed Participants among Disability Subgroups, Odds Ratio (OR), and 95% Confidence Interval (CI).

Feature	% of Highly agree with the ease of use telehealth		OR	95% CI	
	Disabled (%)	Non-Disabled (%)		LB	UB
**Background Characteristics**					
**Sex**					
Female	62.5	74.0	0.58	0.24	1.40
Male	44.4	82.0	0.17***	0.04	0.74
**Age**					
< 25	55.6	73.1	0.34**	0.12	1.01
25–34	66.7	79.7	0.80	0.21	3.12
35+	50.0	86.0	0.15**	0.02	1.03
**Educational level**					
High school or less	37.5	67.5	0.29**	0.10	0.87
University and above	76.5	80.9	0.77	0.24	2.46
**Place of residence**					
Rural	–	50.0	–	–	–
City	57.6	77.5	0.39**	0.19	0.82
**Explanatory Variables**					
**Previous experience with a telehealth service**					
Experienced	72.7	78.4	0.73	0.18	2.98
Inexperienced	50.0	75.9	0.32***	0.73	0.77
**Easiness of using the Internet**					
Not Easy	44.4	44.0	1.02	0.21	4.3
Very Easy	62.5	78.9	0.45*	0.19	1.06
**Chronic Disease Status**					
Have a chronic disease	50	76.5	0.31*	0.07	1.30
Don’t have a chronic disease	61.9	76.7	0.49	0.20	1.23
**N**	**57.6**	**76.7**	0.41**	**0.20**	**0.86**

Chi-square test of independence significance results: ***(<0.01), **(<0.05), *(<0.1).

**Fig 2:**
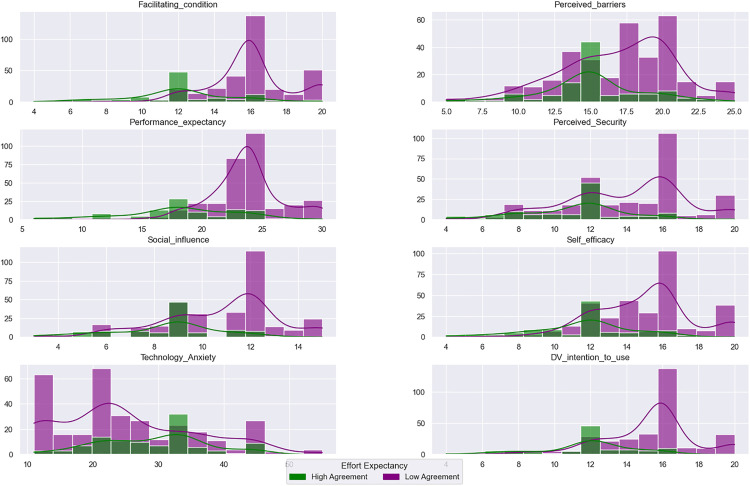
Frequency and Density plot of features Across Classes of Ease of using the telehealth system “Effort Expectancy”.

To fully understand the distribution, spread, and density of the telehealth-related continuous factors, Fig 2 depicts the histogram plot of each factor as well as the density plot. Further, to visualize the influence of each factor on the binary outcome, the plot is produced across classes of the target variable. Although the sample of individuals who have low agreement is lower than those who highly agree, the figure shows different distributions of all factors across the two subgroups. Density plots show a bimodal pattern for most factors, which support the discretization implemented into a binary scale.

Additionally, for most factors, the highly agreed class shifted to the right, indicating a larger rating for all factors than the low agreed class. This summarizes the significant effect of telehealth-related factors on the target variable. However, for the “Technology Anxiety” factor, the subgroup with high agreement is shifted to the left, indicating a lower rating compared to the subgroup with low agreement. This makes sense as individuals with perceptions coinciding with the ease of using telehealth have larger disagreement rates with perceptions about encountering anxiety with using technology.

To fit and validate the classification models, data is segmented into a training set and a testing set with a ratio of 70% to 30%. Thus, out of 428 Saudi individuals, 299 individuals were chosen for the training set, and 129 were chosen for the test set. To optimize the hyperparameters of the RF model while maintaining control of overfitting, we implement the 5-fold cross-validation procedure within a grid search framework. The study suggests tuning the hyper-parameters in the settings so as to choose: the number of trees in the forest as 1000, 2000, and 3000, the maximum depth of the tree as 100, 200, and 300, the minimum size of the leaf as 1, 2, and 4, and finally, the minimum number of samples to split an internal node as 2, 3, and 5. This resulted in tuning 3^4 ^= 81 hyperparameters and training 405 models. The training dataset was partitioned into five equal folds, where for each hyperparameters’ combination, the model was fitted iteratively on four folds (serving as training) and validated on the remaining fold (serving as testing). This process was repeated five times to ensure each fold served as validating or testing fold. The final accuracy scores for each hyperparameters’ combination were then computed as the average across the 5 iterations of the testing folds. Parameter selection via this approach, which does not rely solely on the training dataset or a single validation fold, will imply robust and generalizable predictive estimates and ensure preventing overfitting [15]. The best parameters chosen to fit the Random Forest model are those that give the highest training score and the highest test accuracy. Their values are: 1000 trees in the forest, with 100 max depths, 2 minimum number of samples to split an internal node, and 2 the minimum size of the leaf (terminated node).

From the perspective of prediction accuracy, Table 3 displays the accuracy measures resulting from the training and testing datasets for both models. Generally, all accuracy measures of the test results are lower than that of training results in both models. Accuracy measures of RF are slightly higher than that of LR in both datasets, which reflects a higher predictive performance of the RF model. However, in real-world applications, higher predictivity is translated into more accurate data-driven policy decisions, especially when it is related to allocating resources to improve and develop telehealth systems.

**Table 3:** Accuracy Results from Random Forest and Binary Logistic Regression Models by Data Types.

Models Accuracy Metrics	Random Forest		Binary Logistic	
**Data**	**Train**	**Test**	**Train**	**Test**
**TN**	53	24	51	24
**FN**	**5**	**11**	12	14
**FP**	**17**	12	19	12
**TP**	224	82	217	79
**Sensitivity**	**0.978**	**0.882**	0.948	0.849
**Specificity**	**0.757**	0.667	0.729	0.667
**Precision**	0.929	0.872	0.919	0.868
**F1-Score**	**0.953**	**0.877**	0.936	0.859
**Overall Accuracy**	**0.926**	**0.822**	0.896	0.798
**G-mean**	**0.861**	**0.767**	0.831	0.753
**ROC – AUC**	**0.868**	**0.774**	0.838	0.758
**C-Index**	**0.981**	**0.876**	0.936	0.862

**Fig 3:**
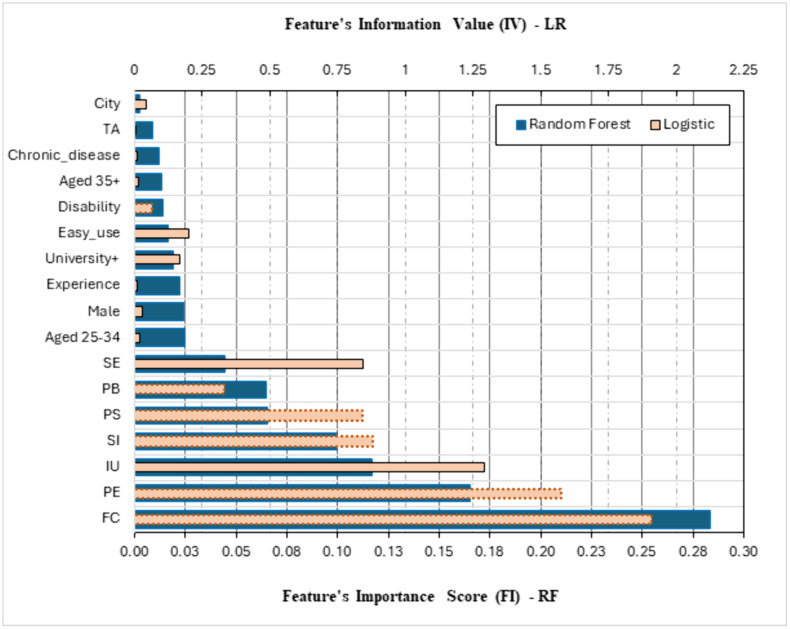
Information Value Scores (Binary Logistic Regression) and Feature Importance Scores (Random Forest) Across Features (for IVs from LR, a bar with dashed border indicates a significant feature in the binary logistic model).

Both Binary Logistic and Random Forest models are fitted on the training dataset. Fig 3 depicts the feature information values (IV) as produced by LR model and the feature importance (FI) scores as produced by RF. Since the scale of the two metrics is highly different, each measure is presented on a separate axis such that FIs are on the primary bottom axis and IVs are on the secondary upper axis. Bars in the plot are ranked in descending order by FI from the RF model.

Both models show that the telehealth related factors are the most contributing features in predicting Effort Expectancy except for Self-Efficacy (SE) and Perceived Barriers (PB) where PB has a low contribution by lower IV, although it has a highly significant effect (a dashed border bar) on Effort Expectancy, inferred by p = 0.02 in Table 4. Based on LR, most of those features show a significant effect, as their associate p < 0.05 in Table 4 (indicated by dashed border bars). In both models, City and Technology Anxiety (TA) have the least contribution, while Disability, easy internet usage and ‘University and above’ education level have a moderate contribution. Male individuals in the middle age group with previous experience in using eHealth show moderate contribution based on RF, while they do not based on LR.

**Table 4:** Logistic Classification Model of the Effort Expectancy.

Model Features	Coefficient	Odds Ratio	P-Value	Lower 95% CI	Upper 95% CI
Const	−5.226	0.005	0.003	−8.662	−1.791
Aged, 25–34	−0.111	0.895	0.844	−1.211	0.990
Aged 35+	−0.009	0.992	0.992	−1.671	1.654
Male	0.823	2.278	0.137	−0.262	1.908
University+	−0.297	0.743	0.592	−1.386	0.791
Chronic_disease	−0.269	0.764	0.722	−1.750	1.212
Disability**	−1.618	0.198	0.037	−3.139	−0.097
Experience	−0.526	0.591	0.328	−1.580	0.529
Easy_use	0.210	1.234	0.787	−1.317	1.737
City*	2.751	15.653	0.062	−0.138	5.640
SE	0.032	1.032	0.953	−1.011	1.074
FC***	2.218	9.186	0.000	1.133	3.303
PB**	1.287	3.623	0.024	0.167	2.408
PE***	1.548	4.701	0.006	0.437	2.659
PS**	1.203	3.329	0.044	0.032	2.374
SI***	1.339	3.817	0.016	0.246	2.433
TA	−1.141	0.319	0.176	−2.794	0.512
IU	0.589	1.803	0.297	−0.518	1.696

***(<0.01), **(<0.05), *(<0.1).

Both models coincide with pointing out the most contributing features in predicting the target variable. The odds ratio for the significant features presented in Table 4 can be used to interpret the impact of those factors on the response. Ranking significant features by their associated p-value shows the rank to be FC, PE, SI, PB, Disability, and PS, which exactly coincides with their rank based on the feature importance scores from RF model, except for Disability, which has the lowest moderate FI score.

Table 4 shows that perceptions supporting the easiness of using telehealth services decrease for disabled individuals by 80% (p = 0.04) compared to non-disabled individuals. Further, insights towards the ease of using telehealth systems are 9 times (p < 0.005) for individuals having higher perceptions of the availability of facilitating conditions (FC) than those who have lower perceptions. Furthermore, participants in favor of telehealth being helpful and increasing service quality (PE) are 4.7 times (p = 0.006) more likely to have a higher perception towards the ease of using the telehealth system than those who are not in favor.

Additionally, individuals who highly support security (PS) with using and exchanging personal information via telehealth systems are 3.3 times (p = 0.04) more likely to support the ease of using telehealth services than those who do not. Also, participants with higher beliefs of the impact of friends and health practitioners (SI) on their use of telehealth services are 3.8 times (p = 0.016) more likely to support the easy usage of telehealth systems. Finally, individuals who do not support that “the existence of barriers (PB) hinders telehealth usage” are 3.6 times (p = 0.024) more likely to highly agree with the ease of using telehealth services than those who support.

## Discussion

This paper aimed to explore the impact of disability, along with other factors, on telehealth usage, examining the degree of ease people feel while using telehealth services in Saudi Arabia. The paper utilized the Binary Logistic Regression (LR) and the Random Forest algorithm (RF) to predict the participants’ attitudes towards the ease of using Telehealth services.

The main results of the descriptive analysis indicated that individuals with perceptions coinciding with the ease of using telehealth have lower anxiety about using technology. Both analysis models showed that the telehealth-related factors (Facilitating Condition, Perceived Barriers, Intention to Use Telehealth system, Social Influence, Perceived Security, Performance Expectancy, Self-Efficacy, and Technology Anxiety) are the most contributing features to predict the easiness of using telehealth systems. However, Self-Efficacy and Perceived Barriers where Perceived has a low contribution.

The interface quality and the ease of use are very crucial for user utilization and engagement of telehealth systems [19,20]. Besides, perceptions are the main driver of technology use and acceptance; the higher ease and increased accessibility, the higher positive perceptions the user has [19,21].

Self-efficacy, including confidence in using telehealth systems, alongside the system’s ease of use and perceived usefulness, boosts trust in the system and supports participation, which in turn strongly impacts the technological acceptance and the ease of use [21].

The social capital comprises the social trust, norms, and networks [22]; not only affects the perceived ease of use and the perceived usefulness of the system positively, but also impacts the spread of health information, and the health awareness norms, with higher accessibility to health services, and the social network support of patients [23]. Additionally, social capital positively impacts the perceived ease of use and perceived usefulness [21]. Main findings show that disabled respondents across various demographic subgroups in Saudi Arabia are significantly less likely than their non-disabled counterparts to perceive telehealth services as easy to employ. This gap is remarkably noticed among disabled participants who are older, males, have lower levels of education, have chronic diseases, or lack any prior experience using telehealth services.

The literature addressed the disparities between disabled and non-disabled patients’ perceptions; in the case of patients with disability, the use of telehealth services depends mainly on the digital literacy of caregivers, the acceptance of telehealth, the differences in treatment methods, the cost, and the quality of care in nursing homes (in case of elderly patients) [24]. Additionally, Liu et al. (2025) studied telehealth use among patients with hearing loss or hearing difficulties; they concluded that those patients have the same rate of telehealth use as the general population [25]. Yet, they experience significant barriers regarding platform accessibility, ease of usability, and convenience, which recommends more support for disabled patients, and better platform accessibility and features enhancement for easier utilization.

These results coincide with Schlottke et al. (2024) and Mizana et al. (2023), ensuring that supportive factors enhance telemedicine adoption, while barriers have minimal impact [26,27]. Additionally, in both models, City and Technology Anxiety have the least contribution. Barriers such as lack of technology literacy or access could negatively impact the intention to use telehealth systems [28]. Nevertheless, Disability, easy internet usage, and ‘University and above’ education level have a moderate contribution to telehealth use. This study concluded that disability reduces the easiness of telehealth use, which contradicts Xie et al. (2023) who concluded that patients with moderate or severe disability are more likely to use telehealth systems [29]. Xie et al. (2023) showed that the population with severe disability and multiple disabilities have a higher propensity to use telehealth due to poor health status and higher needs for health services [29]. The patients with mobility complications got used to telehealth services before the pandemic, and after the pandemic, they adapted quickly to these services because they had experienced them before. Moreover, the disabled patients with higher educational attainment, living in urban areas, and having insurance coverage have higher use of telehealth services regardless of the severity of their disability. The study recommended further research for disability subgroups and the telehealth use differentials regarding their type and severity of disability [29].

Additionally, Choi et al. (2023) surveyed U.S. adults ages 51 and older, and they concluded that cognitive and visual impairments are associated with higher use of telehealth than in-person visits (it is better to use a combination of health care types) [30]. On the other hand, people with three or more disabilities are more likely to have in-person visits alone. Also, the study recommended that tele-health services should be followed up with in-person visits, especially among elderly patients. Moreover, they suggested platforms with audio features to support patients with visual impairments.

Regarding the demographic variables, Male individuals in the middle age group with previous experience in using eHealth show moderate contribution. Hence, the higher the patient’s age, the lower the willingness to use telehealth, but the sex didn’t show a significant impact on telehealth use in the literature [31].

The main limitations of this study was the small sample size, which required caution in our analysis and the usage of the appropriate statistical methodologies. Additionally, the study relied on self-reported data, which may have biased responses due to personal estimates or recall bias. The questionnaire was distributed electronically via social media, potentially introducing sample selection bias by excluding individuals who do not use these platforms or have limited internet access. Therefore, the study findings may not be fully generalizable to all segments of Saudi society, especially those underrepresented in the sample. Nevertheless, this study estimated the impact of key factors influencing telehealth use in Saudi Arabia which wasn’t covered in the previous research. It also highlights the contribution of these factors to the utilization of telehealth services.

For future research, other factors that might affect the easiness of using telehealth systems can be taken into consideration, especially according to health workers’ opinions, as health workers are the main persons who face difficulties in implementing telehealth. Moreover, other factors can affect the use of telehealth on the macro level such as government initiatives like Vision 2030, technological advancements, stakeholder support and investment [32], suggesting the implementation of multilevel analysis with data from multiple countries.

Based on our findings, to have a more inclusive Saudi telehealth system, policymakers should take into consideration the cultural differences and the different needs of patients. Moreover, include easier accessibility features to guarantee the ease of use for telehealth platforms. Nevertheless, boosting digital literacy among the population, especially the elderly, can facilitate the process of using the telehealth system. Finally, reinforcing the telehealth infrastructure with secure systems and high-speed data transfer is crucial for adopting the telehealth system.

## Conclusion

This study explored the factors that influence perceptions of the ease of use of telehealth services in Saudi Arabia, with a particular focus on disabled individuals. The findings showed that disability is associated with significantly lower perceptions of ease of use of health services. Our study also confirmed the key role of facilitating conditions, performance expectancy, social influence, and perceived safety in promoting positive adoption of telehealth. Our study highlights the importance of formulating more inclusive and equitable policies to considers needs disabled persons.

### Declarations

#### Consent for publication.

Informed consent from participants aged 18 years and older. The participants should answer a question of voluntary participation to be directed to the survey. Anonymity and confidentiality were guaranteed and maintained.

## Supporting information

S1 File Random Forest Framework. (a) Illustration of RF model construction using bootstrapped samples and feature subsets. (b) Diagram of prediction process via majority voting. Full explanation is included in Supplementary File 2.
(ZIP)

S2 File ROC Curve and Classification Thresholds [33–36]. ROC curve showing sensitivity vs. false positive rate with threshold examples. Explained in detail in Supplementary File 2.
(TIF)

S3 File Telehealth. (SAV)

S4 File Importing all required Python packages. (PDF)
