# Supplementary figures and images for: Perceived ease of use of telehealth services and associated factors in Saudi Arabia: A cross-sectional study
Source: PLoS One. 2025 Oct 29;20(10):e0334943. doi: 10.1371/journal.pone.0334943 (PMC12571244; doi:10.1371/journal.pone.0334943)

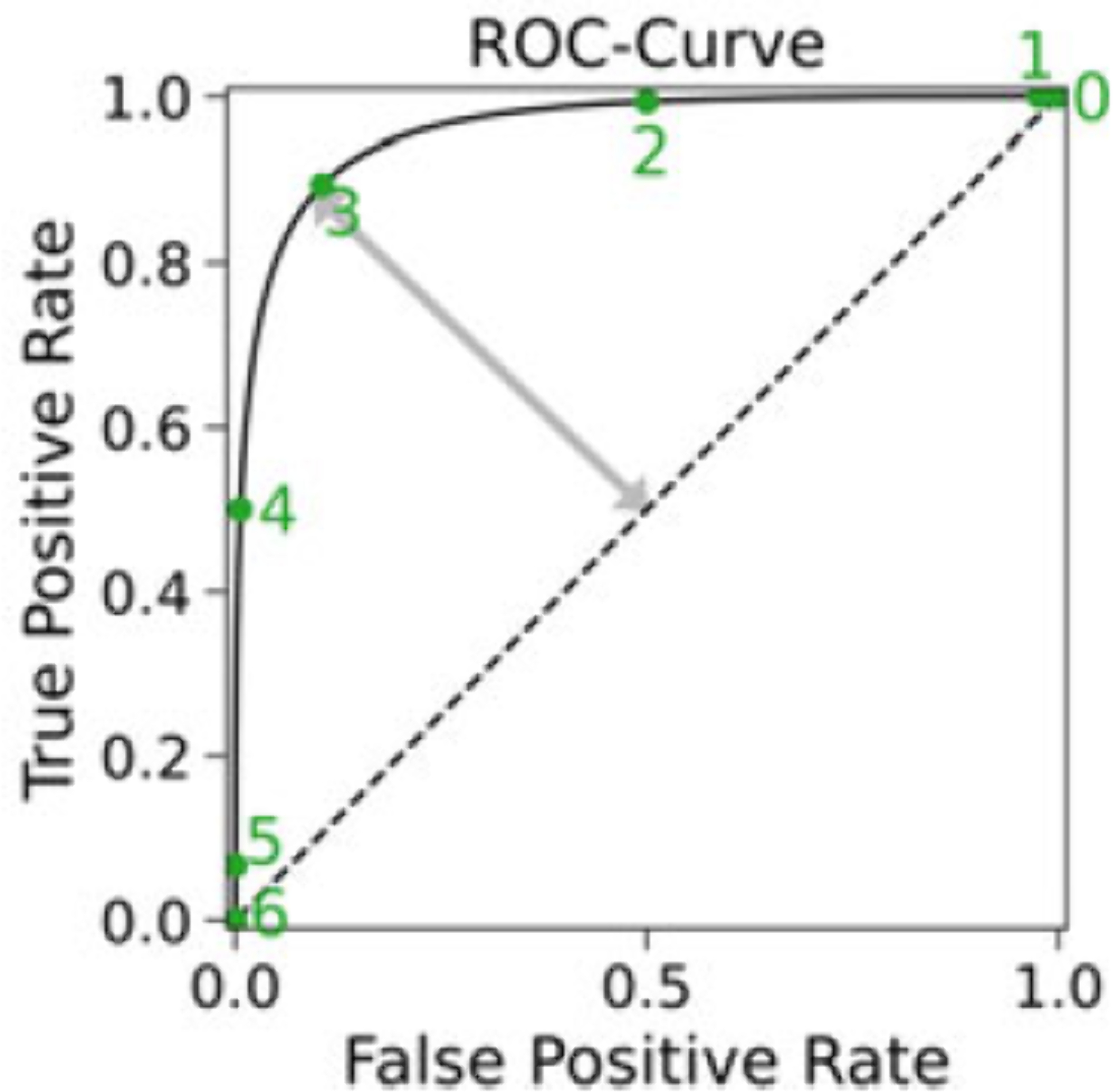

Supplement: S2 File — ROC curve showing sensitivity vs. false positive rate with threshold examples. Explained in detail in Supplementary File 2. (TIF) [file pone.0334943.s002.tif]
