# Supplementary material for: Perceived ease of use of telehealth services and associated factors in Saudi Arabia: A cross-sectional study
Source: PLoS One. 2025 Oct 29;20(10):e0334943. doi: 10.1371/journal.pone.0334943 (PMC12571244; doi:10.1371/journal.pone.0334943)
Supplement: S4 File — (PDF) [file pone.0334943.s004.pdf]

```
## Importing all required Python packages
import pandas as pd
import numpy as np
import matplotlib.pyplot as plt
import seaborn as sns
from scipy.stats import gaussian_kde
from scipy.stats import chi2_contingency
from sklearn.linear_model import LogisticRegression
import statsmodels.api as sm
from sklearn.model_selection import train_test_split
from sklearn.metrics import classification_report, accuracy_score
from sklearn.metrics import confusion_matrix, roc_curve, auc,
roc_auc_score
import lifelines
from lifelines.utils import concordance_index
from sklearn.ensemble import RandomForestClassifier



## Set the working directory
import os
#os.chdir("YourPath")
os.chdir("D:/Niveen Wrking Files/Post-PhD Researches/My Own
researches/Research 25")
data = pd.read_csv("Telehealth_SA.CSV")

## Figure 1
EE = data['Effort_expectancy']
plt.figure(figsize=(10, 6))
kde = gaussian_kde(EE)
kde.set_bandwidth(bw_method=0.35)
x_range = np.linspace(EE.min(), EE.max())
density = kde(x_range)
plt.plot(x_range, density, 'b-', linewidth=2, label='KDE')
plt.fill_between(x_range, density, alpha=0.1, color='blue')
plt.xticks(np.arange(4, 22, 2))
plt.yticks(np.arange(0, 0.22, 0.05))
plt.xlabel('Effort Expectancy Scores', fontsize=12)
plt.ylabel('Density', fontsize=12)
plt.grid(True, alpha=0.8)
plt.legend("")
plt.tight_layout()
plt.show()
plt.close()

# Table 1
features = ['male_labeled', 'age_class', 'city_labeled',
            'educ_labeled', 'Dis_labeled','Exp_labeled',
            'easy_labeled', 'disease_labeled']
dep_var = 'Ee_labeled'

result_tables = []

for feature in features:
    contingency_table = pd.crosstab(data[feature], data[dep_var])
    chi2, p, dof, expected = chi2_contingency(contingency_table)

    table = (data.groupby(feature, observed=True)[dep_var]
               .value_counts(normalize=True)
               .mul(100)
               .unstack())
```

```
    row_totals = data.groupby(feature,
observed=True)[dep_var].count()
    table['Total_n'] = row_totals

    table.columns = table.columns.astype(str)
    table.insert(0, 'Feature', feature)
    table.insert(1, 'Category', table.index)
    table.insert(2, 'Chi2 p-value', p)

    result_tables.append(table.reset_index(drop=True))

final_table = pd.concat(result_tables, ignore_index=True)
final_table = final_table.reset_index(drop=True)

final_table

# Overall total & Percentage for Effort_Expectancy (be added to Table
1)
value_counts = data['Ee_labeled'].value_counts()
percentages = data['Ee_labeled'].value_counts(normalize=True) * 100
result = pd.DataFrame({
    'Frequency': value_counts,
    'Percentage': percentages.round(1)})

result

# Table 2
pd.pivot_table(data=data,
               index='male_labeled',
               columns='Dis_labeled',
               values='Ee_bin',
               aggfunc='mean',
               margins=True,
               margins_name='Totals')\
              .round(3)*100
pd.crosstab(data['male_labeled'], data['Dis_labeled'], margins=True)

pd.pivot_table(data=data,
               index='age_class',
               columns='Dis_labeled',
               values='Ee_bin',
               aggfunc='mean',
               margins=True,
               margins_name='Totals')\
              .round(3)*100
pd.crosstab(data['age_class'], data['Dis_labeled'], margins=True)

pd.pivot_table(data=data,
               index='educ_labeled',
               columns='Dis_labeled',
               values='Ee_bin',
               aggfunc='mean',
               margins=True,
               margins_name='Totals')\
              .round(3)*100
pd.crosstab(data['educ_labeled'], data['Dis_labeled'], margins=True)

pd.pivot_table(data=data,
               index='city_labeled',
               columns='Dis_labeled',
```

```
               values='Ee_bin',
               aggfunc='mean',
               margins=True,
               margins_name='Totals')\
              .round(3)*100
pd.crosstab(data['city_labeled'], data['Dis_labeled'], margins=True)

pd.pivot_table(data=data,
               index='disease_labeled',
               columns='Dis_labeled',
               values='Ee_bin',
               aggfunc='mean',
               margins=True,
               margins_name='Totals')\
              .round(3)*100
pd.crosstab(data['disease_labeled'], data['Dis_labeled'],
margins=True)

pd.pivot_table(data=data,
               index='easy_labeled',
               columns='Dis_labeled',
               values='Ee_bin',
               aggfunc='mean',
               margins=True,
               margins_name='Totals')\
              .round(3)*100
pd.crosstab(data['easy_labeled'], data['Dis_labeled'], margins=True)

pd.pivot_table(data=data,
               index='Exp_labeled',
               columns='Dis_labeled',
               values='Ee_bin',
               aggfunc='mean',
               margins=True,
               margins_name='Totals')\
              .round(3)*100
pd.crosstab(data['Exp_labeled'], data['Dis_labeled'], margins=True)

# Figure 2
Cont_var = data[['Facilitating_condition','Perceived_barriers',
       'Performance_expectancy', 'Perceived_Security',
'Social_influence',
       'Self_efficacy', 'Technology_Anxiety', 'DV_intention_to_use']]
colors = {0: 'green', 1: 'purple'}
plot = fig, axes = plt.subplots(4, 2, figsize=(15, 10))

for i, var in enumerate(Cont_var):
    row, col = divmod(i, 2)
    sns.histplot(data=data, x=var, hue='Ee_bin', palette=colors,
kde=True,
                 ax=axes[row, col], bins=15)
    axes[row, col].set_title(f'{var}')
    axes[row, col].set_xlabel("")
    axes[row, col].set_ylabel("")
    axes[row, col].legend("")
    axes[row, col].grid(True)

plt.tight_layout(rect=[0, 0, 1, 0.95])
plt.subplots_adjust(wspace=0.3, hspace=0.3)
plt.show()
```

```
# Results for Table 3
# Predective Models
features_Ee = ['med_age',
'old_age','male','University+','Chronic_disease',

'Disability','E_expereince','easy_use_binary','city','SE_bin',
               'FC','PB','PE','PS','SI','TA','IU']

X = data[features_Ee]
y = data['Ee_bin']

X_train, X_test, y_train, y_test = train_test_split(X, y,
test_size=0.3,
                                                     random_state=0)

columns = X_train.columns
y_train = y_train.values.ravel()

# Logistic Regression (Model 1)
logist=LogisticRegression()
cols = ['med_age', 'old_age','male','University+','Chronic_disease',

'Disability','E_expereince','easy_use_binary','city','SE_bin',
        'FC','PB','PE','PS','SI','TA','IU']

# Figure 3 (Information Values)
def IV_calc(data,var):
    dataf = data.groupby([var])['Ee_bin'].agg(['count','sum'])
    dataf.columns = ["Total","bad"]
    dataf["good"] = dataf["Total"] - dataf["bad"]
    dataf["bad_per"] = dataf["bad"]/dataf["bad"].sum()
    dataf["good_per"] = dataf["good"]/dataf["good"].sum()
    dataf["I_V"] = (dataf["good_per"] - dataf["bad_per"])
    * np.log(dataf["good_per"]/dataf["bad_per"])
    return dataf

Iv_list = []
for col in cols:
    assigned_data = IV_calc(data = data,var = col)
    iv_val = round(assigned_data["I_V"].sum(),3)
    dt_type = data[col].dtypes
    Iv_list.append((iv_val,col,dt_type))
Iv_list = sorted(Iv_list,reverse = True)
for i in range(len(Iv_list)):
    print (Iv_list[i][0],",",Iv_list[i][1],",type =",Iv_list[i][2])

print ("Accuracy Report (Train Sample)")
y_pred = logist.predict(X_train)
cm_df = pd.DataFrame(confusion_matrix(y_train, y_pred),
                    index=['Actual Negative', 'Actual Positive'],
                    columns=['Predicted Negative', 'Predicted
Positive'])
cm_df

class_dict = classification_report(y_train, y_pred, output_dict=True)
class_df = pd.DataFrame(class_dict).T
class_df=class_df.round(3)
class_df
accuracy_score(y_train,y_pred).round(4)
```

```
logist.score(X_train,y_train).round(4)
roc_auc_score(y_train,y_pred).round(4)
y_scores = logist.predict_proba(X_train)[:, 1]
c_index = concordance_index(y_train, y_scores)
c_index.round(4)

print ("Accuracy Report (Test Sample)")
y_pred = logist.predict(X_test)
cm_df = pd.DataFrame(confusion_matrix(y_test, y_pred),
                    index=['Actual Negative', 'Actual Positive'],
                    columns=['Predicted Negative', 'Predicted
Positive'])
cm_df
class_dict = classification_report(y_test, y_pred, output_dict=True)
class_df = pd.DataFrame(class_dict).T
class_df=class_df.round(3)
class_df
accuracy_score(y_test,y_pred).round(4)
logist.score(X_test,y_test).round(4)
roc_auc_score(y_test,y_pred).round(4)
y_scores = logist.predict_proba(X_test)[:, 1]
c_index = concordance_index(y_test, y_scores)
c_index.round(4)

# Random Forest (Model 2)
rf_fit = RandomForestClassifier(n_estimators=1000,
                                criterion="gini",
                                max_depth=100,
                                min_samples_split=2,
                                min_samples_leaf=2,
                                random_state=42)

rf_fit.fit(X_train, y_train)

print ("Accuracy Report (Train Sample)")
y_pred = rf_fit.predict(X_train)
cm_df = pd.DataFrame(confusion_matrix(y_train, y_pred),
                    index=['Actual Negative', 'Actual Positive'],
                    columns=['Predicted Negative', 'Predicted
Positive'])
cm_df
class_dict = classification_report(y_train, y_pred, output_dict=True)
class_df = pd.DataFrame(class_dict).T
class_df=class_df.round(3)
class_df
accuracy_score(y_train,y_pred).round(4)
rf_fit.score(X_train,y_train).round(4)
roc_auc_score(y_train,y_pred).round(4)
y_scores = rf_fit.predict_proba(X_train)[:, 1]
c_index = concordance_index(y_train, y_scores)
c_index.round(4)

print ("Accuracy Report (Test Sample)")

y_pred = rf_fit.predict(X_test)
cm_df = pd.DataFrame(confusion_matrix(y_test, y_pred),
                    index=['Actual Negative', 'Actual Positive'],
                    columns=['Predicted Negative', 'Predicted
Positive'])
cm_df
class_dict = classification_report(y_test, y_pred, output_dict=True)
```

```
class_df = pd.DataFrame(class_dict).T
class_df=class_df.round(3)
class_df
accuracy_score(y_test,y_pred).round(4)
rf_fit.score(X_test,y_test).round(4)
roc_auc_score(y_test,y_pred).round(4)
y_scores = rf_fit.predict_proba(X_test)[:, 1]
c_index = concordance_index(y_test, y_scores)
c_index.round(4)

# Figure 3 (Feature Importance)
importances = rf_fit.feature_importances_
std = np.std([tree.feature_importances_ for tree in
rf_fit.estimators_], axis=0)
indices = np.argsort(importances)

colnames = list(X_train.columns)
sorted_features = [colnames[i] for i in indices]

print("\nFeature ranking:\n")
for i in range(len(indices)):
    feature_importance_df = pd.DataFrame({
    'Feature': sorted_features,
    'Importance': importances
})
feature_importance_df =
feature_importance_df.sort_values(by='Importance',

ascending=False)
feature_importance_df

# Table 4
data['med_age'] = np.where(
    data['age_class'].str.contains("Medium").fillna(False),1,0)
data['med_age'].dtype
data['old_age'] = np.where(
    data['age_class'].str.contains("Old").fillna(False),1,0)
data['old_age'].dtype

features_Ee = ['med_age',
'old_age','male','University+','Chronic_disease',

'Disability','E_expereince','easy_use_binary','city','SE_bin',
               'FC','PB','PE','PS','SI','TA','IU']

logit_model=sm.Logit(y_train,sm.add_constant(X_train))
result=logit_model.fit()
odds_ratios = pd.DataFrame({
    "Coefficient": result.params,
    "Odds Ratio": np.exp(result.params),
    "P-Value": result.pvalues,
    "Lower 95% CI": result.conf_int()[0],
    "Upper 95% CI": result.conf_int()[1]
})

odds_ratios
```
